# Comparison of Effects of Different Sacrificial Hydrogen Bonds on Performance of Polyurethane/Graphene Oxide Membrane

**DOI:** 10.3390/membranes12050517

**Published:** 2022-05-13

**Authors:** Wen Fu, Li Wang, Zhuohang Huang, Xiaoyan Huang, Zhijin Su, Yixing Liang, Zhitin Gao, Qingyu Pan

**Affiliations:** 1 College of Material Science, Guangdong University of Petrochemical Technology, Maoming 525000, China; a449192213@gdupt.edu.cn (W.F.); a2156947252@163.com (Z.H.); a314598691@163.com (X.H.); a3481857608@163.com (Z.S.); a1412843452@163.com (Y.L.); a449192213@163.com (Z.G.); a390375448@163.com (Q.P.); 2 College of Chemical Engineering, Guangdong University of Petrochemical Technology, Maoming 525000, China

**Keywords:** graphene oxide, different number-average molecular polyethylene glycols, polyurethane, organic-inorganic hybrid membranes, hydrogen bonds, properties

## Abstract

Processing robust mechanical properties is important for elastomeric materials. In this work, different molecular weights of polyethylene glycols (PEG) were used to modify graphene oxide (GO) in order to study the relationship between the number of hydrogen bonds and the properties of the polyurethane/graphene oxide membrane. The fact of PEG was successfully grafted onto the surface of GO was certified by Fourier transform infrared spectra, Raman spectra, X-ray photoelectron spectroscopy. The graft ratio was indicated by thermogravimetric analysis. The presence of hydrogen bonds in PUR/MGO composites membrane was proved by the cyclic loading-unloading test and stress relaxation test. The thermal stability and low-temperature resistance performance of PUR/MGO had been improved compared with PUR/GO. When the molecular weight of PEG grafted on the surface of GO was 600, the tensile strength and elongation at break of the composite membrane were optimal. The reason for the improvement of physical and mechanical properties was that the dispersion of filler in the rubber matrix and the compatibility between filler and rubber had been improved.

## 1. Introduction

Rubber is widely used in the automobile industry, petroleum industry, leather industry, and other fields because of its high wear resistance, high strength, high elasticity, and good aging resistance, etc. For enhanced performance and lifetime of rubber products, fillers such as carbon black (CB) [1], carbon nanotubes (CNTs) [2], carbon nanodots (CDs) [3] and graphene [4], have been introduced into the rubber matrix. Among these nanofillers, graphene is considered to be the most promising one due to its excellent features, such as super Young’s modulus, fracture strength, barrier properties, and conductive properties [5]. However, some articles reported that graphene improved the strength of rubber matrix at the expense of toughness and ductility [6,7]. How to improve the toughness and strength of vulcanizates simultaneously is a challenging issue.

Natural biomaterials are a vast source to inspire us to design robust materials with excellent strength and toughness. For instance, spider silk, bones and shells, they always exhibit a surprising balance of strength, toughness, and ductility [8,9]. Researches have demonstrated that the supramechanical performance of these natural robust materials is actually achieved by using the sacrificial bond and hidden length on the soft-hard interface to obtain high strength, toughness, and ductility [10]. Inspired by these natural biomaterials, it was recently demonstrated that constructing sacrificial bonds and hidden lengths in cross-linked rubber is a good strategy to obtain excellent mechanical performance [11,12,13,14,15]. Although the bond energy of non-covalently sacrificial bonds such as ionic bonds [11], metal bonds [12], π-π conjugate interactions [13], π-cation interactions [14] and van der Waals force (including hydrogen bonds) [15] is not so high, interface load transfer and strength increase can also be achieved through multi-level and multi-quantity reasonable matching of sacrificial bonds [16]. It is noteworthy is that the dissipation of these sacrificial bonds can dissipate additional energy, thus ensuring the toughness and ductility of materials.

Mao [11] constructed sacrificial ionic bonds in the styrene butadiene rubber (SBR)/GO system by adding a kind of pyridyl-containing styrene-butadiene rubber (VPR). The sacrificial ionic bonds were produced between the pyridine group of VPR and the oxygen-containing functional groups of the GO surface. Tang [12] designed sacrificial metal-ligand bonds in VPR, and the tensile strength of the composite membrane reached 27.8 MPa, which was better than that of the VPR/CB hybrid membrane. Zhao [13] introduced tannic acid (TA) as a stabilizer to prepare functionalized graphene (NTGE). When NTGE addition was 1 phr, the tensile strength and tear strength of natural rubber (NR) were increased by 33% and 20% respectively, owing to the dissipation of the π-π conjugate interactions between TA and graphene upon deformation. Berki [14] introduced sacrificial -cation bonds into the NR/graphene composite membrane by adding cetyltrimethyl ammonium bromide (CTAB). When 5% CTAB (related to the amount of graphene) and 1 phr graphene were added to the NR matrix, the tensile strength and elongation at break of the composite membrane were increased by 19.7% and 3.4%. Yin [15] used polyvinylpyrrolidone (PVP) to modify GO and introduce the sacrificial hydrogen bonds into the SBR matrix. The tensile strength, elongation at break, and tear strength of the composite membrane were significantly enhanced by 517%, 16.3%, and 387%, respectively, compared with those of virgin SBR when the modified GO addition was 5 phr.

From the above discussion, it can be seen that the construction of the sacrificial bonds in the rubber system can achieve an improvement in the strength and toughness of the rubber. However, the type and number of the sacrificial bonds are critical. If the stress of the sacrificial bond destruction is too high because too strong or too many sacrificial bonds are constructed in the rubber system, the rubber materials will undergo macroscopic body rupture before the sacrificial bonds have been destroyed during the deformation process. The rubber materials tend to be “brittle fracture”; conversely, if the sacrificial bonds constructed in the rubber system are too weak, the effect of dissipating the strain energy to maintain the toughness of the materials is hard to achieve. Based on the theoretical calculations [17,18,19,20], Bionics generally believes that the best effect can be achieved when the rupture energy of the sacrificial bonds is 0.7 to 0.8 times that of the molecular main strains. Huang [21] utilized triazolinedione (TAD) to construct sacrificial hydrogen bonds in the pre-crosslinked SSBR network. The performance of SSBR was adjusted by adjusting the amount of TAD to control the number of sacrificial bonds. Song [22] used different pyrimidine molecules to construct hydrogen bonds in poly (vinyl alcohol) (PVA). It has been found that the macroscale properties of PVA exhibited a strong dependence on the number of amino (NH_2_) groups in each pyrimidine molecule. Song [23] designed hydrogen bonds using the sulfonate groups in sulfonated styrene-ethylene/butylene-styrene triblock copolymer (SSEBS) and the hydroxyl groups in PVA to prepare the PVA/SSEBS composite films. It has been found that the addition of 10 wt.% SSEBS led to a world-record toughness of 122 J/g without compromising tensile strength. However, a further increase in the content of SSEBS would lead to a rapid decrease in strength and extensibility. A similar trade-off phenomenon was also found in Zhang’s work [24]. So, the type and content of the sacrificial bond can obviously affect the performance of the materials.

In this paper, GO was functionalized with different number-average molecular polyethylene glycols to prepare the graphene oxide modified by polyethylene glycol (MGO). By mechanical blending, MGO was incorporated into polyurethane rubber (PUR) matrix. The different numbers of hydrogen bond sacrificial units were produced at the interface of MGO and PUR in order to study the relationship between the number of hydrogen bonds on a PUR molecular chain and the properties of the composites. The mechanical properties and thermal properties of PUR/MGO hybrid membranes were systematically studied.

## 2. Materials and Methods

### 2.1. Materials

Polyurethane rubber of type HA-5 (polyether-based) was bought from Shanxi Institute of Chemistry Co., Ltd., Shanxi, China. Multilayer graphene oxide (GO) (layers of 6~10, BET surface area of 350~450 m^2^/g, lamellar size of 5~50 μm) was purchased from Tanfeng Co., Ltd., Suzhou, China. Polyethylene glycol (PEG) of type PEG-200, PEG-600 and PEG-1000 (number-average molecular weight of 200, 600 and 1000, respectively) were obtained from Sinopharm Chemical Reagent Co., Ltd., Shanghai, China. 4-dimethylaminopyridine (DMAP), N,N′-dicyclohexylcarbodiimide (DCC), dicumyl peroxide (DCP), zinc oxide (ZnO), stearic acid (SA) and toluene were purchased from Sigma Aldrich, America, all of them were analytical reagent. All materials were used as received.

### 2.2. Sample Preparation

#### 2.2.1. Preparation of Graphene Oxide Modified by Polyethylene Glycol

**Figure 1:**
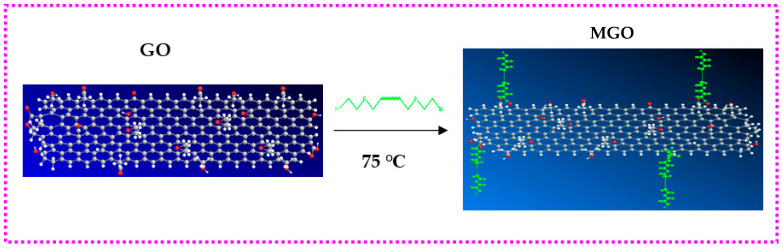
Preparation of polyethylene glycol grafted with graphene oxide.

GO toluene suspension with a concentration of 2 mg/mL was prepared through 30 min ultrasonic treatment. 0.01 mol of PEG, 0.02 mol of DCC and 0.01 mol of DMAP were added to 300 mL of GO toluene suspension and dispersed by ultrasound for 30 min. Then, the content was reacted for 24 h at 75 °C. The graphene oxide modified (MGO) by polyethylene glycol was obtained after filtered by polytetrafluroethylene filter paper, washed with 50 mL of toluene and 700 mL of deionized water in turn, and dried in a vacuum oven at 60 °C. The preparation process was shown in Figure 1. For example, if the number-average molecular weight of PEG used as a modifier was 600, the modified graphene oxide (MGO) was marked as MGO-600.

#### 2.2.2. Preparation of PUR/MGO Composite Membrane

PUR/MGO composite membrane was prepared on a lab scale by the Haake Rheomix 600. The preparation process was briefly described as follows. PUR, GO (or MGO), ZnO and SA were added into the mixing chamber of the Haake Rheomix 600 at 60 rpm and at room temperature (25 °C, similarly hereinafter), and mixed for 4~5 min until a stable torque was achieved. Then DCP was introduced in the mixing chamber and mixed for 2~3 min until the torque reached to equilibrium. Then the composites were taken out from the mixing chamber and put into a double roll mill to mix further. The composites were processed according to the method of triangle-packed for 3 times and thin-passing for 3 times in the double roll mill. Then the composites were parked for 24 h. Finally, the composites were vulcanized at 16 MPa for t_90_ + 2 min at 175 °C to prepare the PUR/MGO composite membrane.

The mass ratio of PUR: GO (or MGO): ZnO: SA: DCP is 100: 2: 1: 0.4: 2. For example, if the filler was MGO-600, the sample was marked as PUR/MGO-600.

### 2.3. Characterizations

Fourier transform infrared spectra (FTIR) were collected on an 8400S FTIR spectrometer (Shimadzu, Japan). The wavenumber range was 4000~400 cm^−1^, the scanning time was 32, and the sample was prepared by potassium bromide troche (KBr) pressing.

Raman spectra were carried out on a LabRAM Aramis Raman microscope (HORIBA Jobin Yvon, France). The excitation wavelength was 532 nm, and the resolution was 1 cm^−1^.

X-ray photoelectron spectroscopy (XPS) measurements were performed on an AxisUltraDLD XPS (Kratos, Britain). The scanning range was 0~1200 eV, the resolution was 0.48 eV, the atomic sensitivity factors of O^1s^ and C^1s^ were 0.733 and 0.314, respectively.

Thermogravimetric analysis (TGA) was carried out on a 209 F3 thermogravimetric analyzer (Netzsch, Germany). The measure range was 35~950 °C for fillers, and the measure range was 50~950 °C for PUR vulcanizates, the heating rate was 30 K/min, the nitrogen was used as the purge gas at a speed of 20 mL/min.

The GO and MGO samples for FTIR, XPS, Raman spectra and TGA were purified using reflux condensation with toluene for 2 h, then washed with 50 mL toluene and dried with a vacuum.

Dynamic mechanical analyses (DMA) were carried out on a 214E dynamic mechanical analyzer (Netzsch, Germany). The mode was stretching. The measurement range was −80~70 °C, the heating rate was 3 K/min, the amplitude was 60 μm, the exciting frequency was 10 Hz. Stress-relaxation tests were also carried out on the 214E dynamic mechanical analyzer.

The tensile properties were tested by a 2080 tensile testing machine (UC, Taiwan). The test standard was GB/T 528-2009, the sample shape was a dumbbell-shaped strip, the thickness of samples was 1.0 mm, the tensile speed was 50 mm/min for cyclic stress-strain measurement, and the tensile speed was 200 mm/min for tensile strength measurement. The fracture energy was calculated by the integral of the stress-strain curve.

The tensile fracture surfaces of PUR vulcanizates were observed on a TM3030 scanning electron microscopy (SEM) (Hitachi, Japan). The acceleration voltage was 15 kV, and the samples were sputtered with platinum.

## 3. Results and Discussion

### 3.1. Interface Structures of GO/PEG

**Figure 2:**
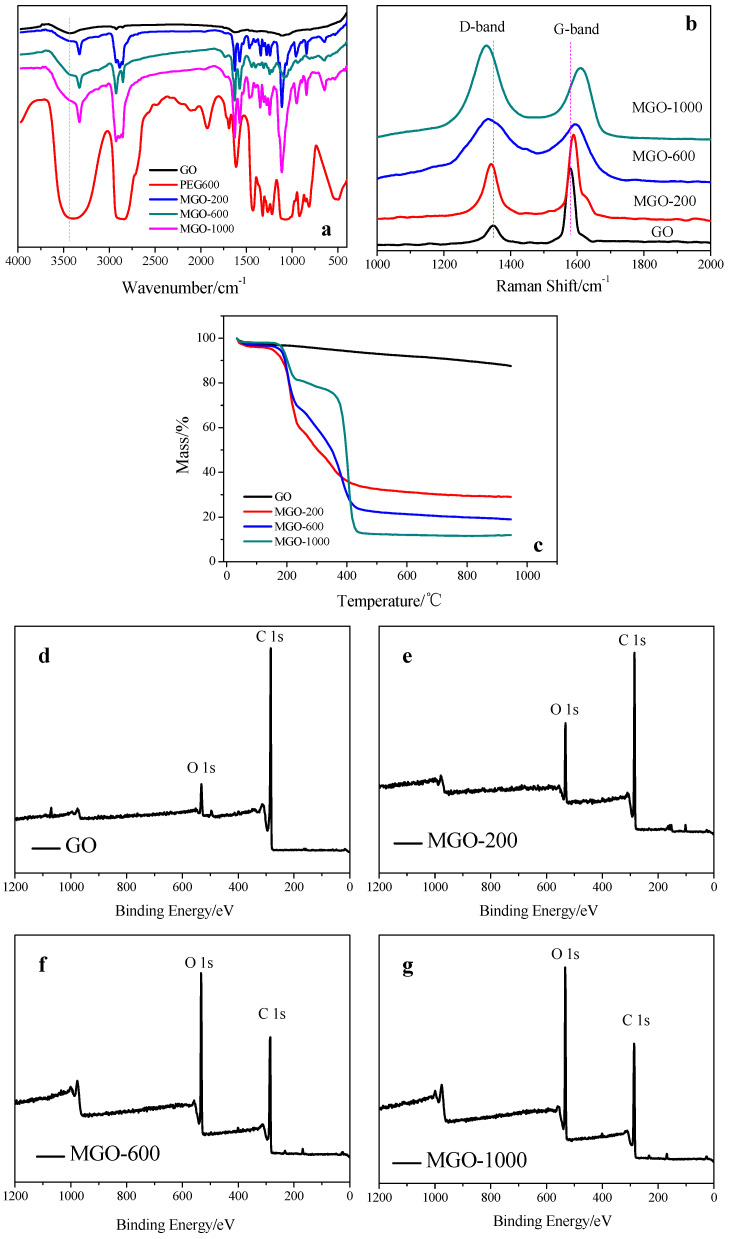
Structural characterizations of GO and MGO. (**a**) FTIR; (**b**) Raman spectra; (**c**) TGA; (**d**–**g**) XPS.

The FTIR spectra of GO, PEG, and MGO samples were shown in Figure 2a. The spectrum of GO showed some typical peaks, corresponding to O-H stretching vibration from -OH and -COOH (3430 cm^−^^1^), C-H stretching vibration (2927 cm^−^^1^ and 2845 cm^−^^1^), C=O stretching vibration from -C=O and -COOH (1734 cm^−^^1^), C=C stretching vibration (1622 cm^−^^1^) and C-O-C stretching vibration (1110 cm^−^^1^) [25]. Meanwhile, the spectra of MGO-200, MGO-600 and MGO-1000 present some changes compared to GO, due to the graft modification of PEG onto the surface of GO. First, the peak intensity of O-H stretching vibration was obviously rising, and the position of the peak was also shifted to lower wavenumber by about 100 cm^−^^1^. This was because that the O-H group was directly attached to the benzene ring in the GO sample, the bond energy of the C-OH in the GO sample was bigger than that of PEG due to the +C action of the benzene ring π-electron. The bigger bond energy could be expressed as the wavenumber of the absorption peak in FTIR spectrum was higher [26]. So the absorption peaks at 3330 cm^−^^1^ of MGO-200, MGO-600 and MGO-1000 were mainly attributed to the O-H stretching vibration from -OH of PEG chains. Second, the peak intensities at 2930~2840 cm^−^^1^, 1640 cm^−1^, 1560 cm^−1^ and 1111 cm^−1^ were stronger compared to GO, and these peaks also existed in pure PEG. The above facts indicate that PEG was grafted onto the surface of GO [25].

The Raman spectra were carried out to investigate the interaction between PEG and GO, and were shown in Figure 2b. It could be found that both GO and MGO presented two characteristic peaks at around 1580 cm^−1^ (G-bond) and 1350 cm^−1^ (D-bond). The G-band was caused by the C-C vibration of a delocalized π electron in graphite, while the D-band corresponded to the vibration of the crystalline edge of graphene oxide, which represented the defect or disorder of graphene oxide [27]. Notably, the D-band’s position of MGO gradually shifted to lower wavenumber and the G-band’s position gradually shifted to higher wavenumber with the increase of the molecular weight of PEG grafted on the surface of GO. For instance, the D-band’s position of MGO-200, MGO-600 and MGO-1000 shifted to lower wavenumber by 9 cm^−1^, 17 cm^−1^ and 24 cm^−1^, respectively, than that of GO, while the G-band’s position shifted to higher wavenumber by 8 cm^−1^, 13 cm^−1^ and 26 cm^−1^, respectively. The reason of these wavenumber shifts was that the interaction between PEG and GO led to electron transfer [28]. In addition, the defect of graphene oxide could be characterized by the intensity ratio of D-band and G-band (I_D_/I_G_), bigger value of I_D_/I_G_ meant more crystal defects [29]. I_D_/I_G_ of GO was 0.30, I_D_/I_G_ of MGO-200, MGO-600 and MGO-1000 were 0.64, 1.06 and 1.30, respectively. The gradually increasing I_D_/I_G_ of MGO was attributed to more PEG grafted onto the surface of GO, causing an increase in the degree of surface defects [29]. The above facts also indicated that PEG was grafted onto the surface of GO.

TGA was used to further measure the graft ratio of PEG on the surface of GO, and was shown in Figure 2c. The TGA curve of GO showed a steady and slow decline with the increase in temperature. However, the TGA curves of MGO showed two ablation platforms with the increase of temperature. The first platform occurred at a temperature of 50 °C to 230 °C, which was mainly due to the loss of adsorbed water in the samples. At a temperature range of 230 °C to 500 °C, the mass loss of GO was 3.4%, which was mainly attributed to the ablation of oxygen-containing groups. However, the mass losses of MGO-200, MGO-600 and MGO-1000 were 30.8%, 48.3% and 69.3%, respectively. These were mainly due to the ablation of PEG. Hence, the graft ratios of MGO-200, MGO-600 and MGO-1000 were around 27.4%, 44.9%, and 65.9%, respectively.

The XPS curves and key data of GO and MGO samples were shown in Figure 2d–g and Table 1. The O^1s^ peak intensity of GO was weak. However, that of MGO was gradually stronger, indicating the incorporation of PEG into the GO system. The O element content of GO was 7.44% and the O/C was only 0.08, which was attributed to the oxygen-containing groups. It indicated that a large number of oxygen-containing functional groups on the surface of GO could be used for the grafting reaction, which provided a guarantee for the subsequent grafting of PEG. A gradual increase in the O element contents and O/C ratios of MGO-200, MGO-600 and MGO-1000 indicated that PEG was successfully grafted onto the surface of GO.

### 3.2. Vulcanization Properties of PUR/MGO Composite Membrane

Vulcanization characteristics are important parameters in rubber processing. The vulcanization characteristics of virgin PUR, PUR/GO and PUR/MGO composite membrane were shown in Table 2. The scorch time (t_10_) and curing time (t_90_) of PUR/GO were longer compared to virgin PUR. It showed that GO could delay the vulcanization of PUR. It was because of a large number of -COOH groups contained on the surface of GO caused GO to exhibit acidic properties. In general, the acidic compounds would delay the vulcanization of rubber [30]. In addition, GO selected for this experiment was prepared by strong acid oxidation. Insufficient washing also caused the raw materials to be acidic, resulting in prolonged vulcanization time. However, the scorch time and curing time of vulcanizates had no significant change when MGO was added compared to virgin PUR. The reason was that the -COOH groups on the surface of GO were reacted, and GO was further washed to remove acidic compounds in the process of preparing MGO.

What’s more, the minimum torque (M_L_) and maximum torque (M_H_) increased when GO and MGO were added. It was due to the introduction of filler-filler and filler-rubber networks in the composites. Generally speaking, the value of M_H_-M_L_ could reflect the cross-link density of the compounds [30]. The cross-link density of PUR/MGO gradually increased with the increase of PEG molecular weight. It was because that part of PEG grafted on the surface of GO could participate in the cross-linking reaction under the action of the DCP vulcanizing agent. The longer and more PEG chains there were, the more likely they were to participate in the cross-linking reaction.

### 3.3. Thermal Stability of PUR/MGO Composite Membrane

**Figure 3:**
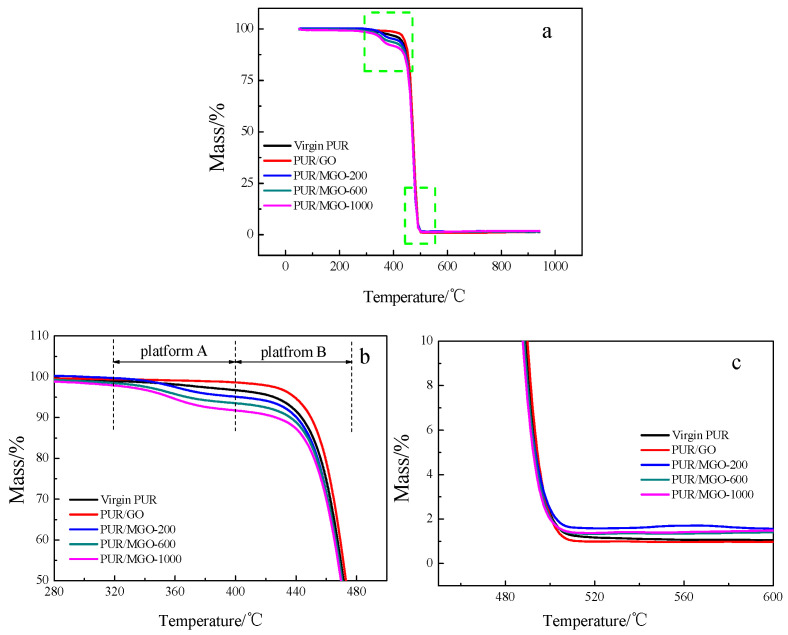
Thermogravimetric analysis of PUR, PUR/GO and PUR/MGO. (**a**) full image; (**b**) partial enlargement of (**a**); (**c**) partial enlargement of (**a**).

The thermal stabilities of virgin PUR, PUR/GO and PUR/MGO vulcanizates were shown in Figure 3 and Table 3. As the temperature increased, the decomposition curves of PUR added with MGO were different from those of GO or virgin PUR. They present two decomposition platforms (Figure 3b). Platform A should be attributed to the erosion of the PEG on the surface of MGO, and platform B was the erosion of the PUR matrix. The initial decomposition temperature (T_i_), maximum decomposition rate corresponding temperature (T_max_) and final decomposition temperature (T_t_) of the virgin PUR vulcanizates were 458.6 °C, 462.1 °C and 472.5 °C, respectively. T_i_, T_max_ and T_t_ increased to 465.2 °C, 468.7 °C and 475.0 °C when 2% GO was added. It was due to the higher decomposition temperature of the filler that would improve the decomposition temperature of the vulcanizates when it was added to the rubber matrix [31]. Besides, GO was a lamellar structure, and a physical fire barrier layer would be formed when the vulcanizates with GO added were ablated. The presence of such a fire barrier layer would slow the escape of decomposition products and subsequently delay further degradation [32].

In addition, for PUR/MGO composites, the T_i_ of platform A decreased from 350.6 °C to 344.7 °C with the molecular weight of PEG increased from 200 to 1000. T_i_ of platform B was less than that of virgin PUR vulcanizates. It was due to the subsequent effect of premature decomposition of PEG. But the T_max_ and T_t_ of PUR/MGO were higher than that of virgin PUR vulcanizates. It was because MGO would be dispersed better after graft modification [33], that stronger filler-rubber interactions and more uniform fire barrier layer were generated, and thus higher thermal stability was achieved. It was also found that the thermal stability of PUR/MGO-1000 was lower than that of PUR/MGO-600. It was because when the filler dispersibility was not the main factor, the effective GO content of PUR/MGO-1000 was less than that of PUR/MGO-600 at the same filler addition because more PEG was grafted for MGO-1000.

### 3.4. Dynamic Mechanical Properties of PUR/MGO Composite Membrane

**Figure 4:**
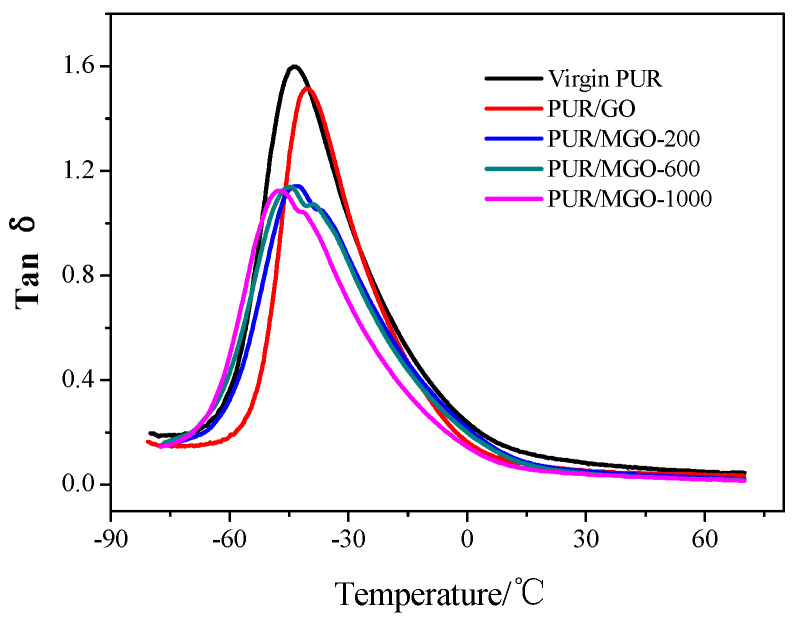
Dynamic mechanical properties of PUR, PUR/GO and PUR/MGO.

The glass transition temperature (T_g_) of the vulcanizates could be measured from the tan δ-temperature curve. The tan δ-temperature curves of virgin PUR, PUR/GO and PUR/MGO were shown in Figure 4. T_g_ of the virgin PUR vulcanizates was −43.8 °C. T_g_ of the vulcanizates increased to −40.2 °C when 2% GO was added, which indicated that the low temperature resistance of the vulcanizates decreased. The reason was that the movement ability of the rubber molecular chains would decrease when GO was added to the rubber matrix because a strong interaction between the GO fillers and the rubber molecular chains would be produced, which would limit the rubber molecular chains to move. This was consistent with the principle that T_g_ of the vulcanizates shifted to a high temperature when the filler such as carbon black was added to the matrix [34].

In addition, the shapes of the tan δ peak changed from their original high and sharp shapes to low and wide shapes when MGO was added. The more interesting thing was that the tan δ peak split into two peaks, which should be related to the existence of multiple movement mechanisms in the molecular chain of PUR/MGO composite membrane. In the dynamic strain process of PUR/MGO composite membrane, besides the movement of the rubber molecular segment, it also includes the destruction and recombination of the hydrogen bonds formed by the rubber molecular chains and PEG chains. This kind of destruction and recombination of the hydrogen bonds would dissipate a portion of the energy, resulting in a change in the shape of tan δ peak.

T_g_ of PUR/MGO-200 composite membrane was −42.6 °C, which was higher than that of virgin PUR vulcanizates. T_g_ of PUR/MGO-600 composite membrane and PUR/MGO-1000 composite membrane were −44.7 °C and −47.4 °C, which were lower than that of virgin PUR vulcanizates. It was because on the one hand, the added MGO would form strongly interaction with the rubber molecular chains, resulting in the movement of the molecular segments to be hindered, thereby causing T_g_ to shift to higher temperature. On the other hand, as mentioned above, the destruction and recombination of the hydrogen bonds would occur in the dynamic strain process of PUR/MGO composite membrane. This movement of the hydrogen bonds was a secondary transition that could be carried out at lower temperature, thereby causing T_g_ to shift to lower temperature. The combined effect of these two offset resulted in the change of the above T_g_. The fact that T_g_ of PUR/MGO composite membrane decreased with the increase of molecular weight of PEG grafted on the surface of GO also indicated that PEG with larger molecular weight would generate more hydrogen bonds in the rubber matrix.

### 3.5. Tensile Properties of PUR/MGO Composite Membrane

**Figure 5:**
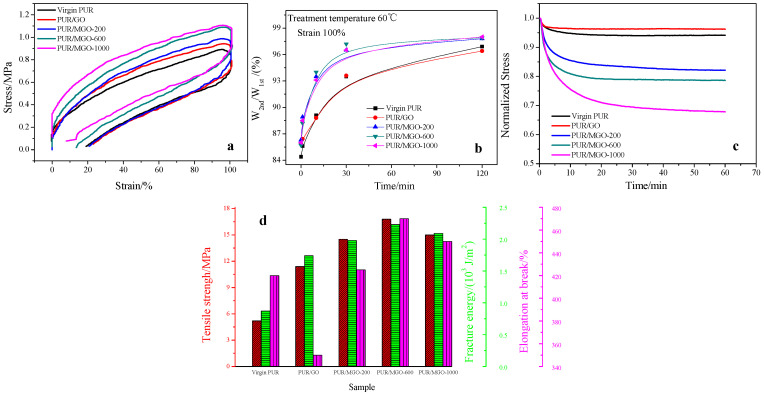
Tensile properties of PUR, PUR/GO and PUR/MGO. (**a**) cyclic loading-unloading; (**b**) hysteresis loss’s recovery; (**c**) stress relaxation; (**d**) mainly mechanical properties.

The presence of sacrificial hydrogen bonds in the PUR matrix could also be demonstrated from the cyclic loading-unloading curves of the membrane. The value of the hysteresis loop was 32.9 a.u. when the virgin PUR vulcanizate was stretched to a predetermined strain of 100% and recovered, and it increased to 38.9 a.u. for PUR/GO (Figure 5a). It was related to the viscoelastic effect of the vulcanizate. The added GO increased the internal friction of the molecular chains during the loading-unloading process, thus increasing the hysteresis loss. In addition, the hysteresis loss continued to rise when MGO was added. The hysteresis loop’s values of PUR/MGO-200, PUR/MGO-600 and PUR/MGO-1000 composite membrane were 40.2, 40.8 and 42.8 a.u., respectively (Figure 5a). It was due to the presence of hydrogen bond in the PUR/MGO system, which was destroyed during the stretching process and produced a large amount of energy dissipation. This was also the reason for the toughness improvement of PUR/MGO composite membrane.

Hydrogen bonds were reversible non-covalent bonds that could be regenerated during the storage or heat treatment. Its recovery was time and temperature dependent. The hysteresis loss’s recovery of virgin PUR, PUR/GO and PUR/MGO composite membrane was shown in Figure 5b. W_1st_ was the first loading-unloading hysteresis loss’s value of the sample, W_2nd_ was the loading-unloading hysteresis loss’s value after the sample was treated for a certain period of time at 60 °C. So the recovery situation of hydrogen bond could be evaluated from the value of W_2nd_/W_1st_. For the virgin PUR and PUR/GO sample, the hysteresis loss gradually recovered with the time, which was due to the re-crimping and re-entanglement of molecular chains, re-generation of the filler-rubber interaction [35]. For PUR/MGO sample, the hysteresis loss quickly recovered in the first 30 min compared to the virgin PUR and PUR/GO sample. The value of W_2nd_/W_1st_ reached to around 96% at 30 min for PUR/MGO, while the value of W_2nd_/W_1st_ only reached to around 93% for the virgin PUR and PUR/GO. It indicated that the hydrogen bonds could be quickly regenerated in the PUR/MGO system.

The stress relaxation tests were carried out to further illustrate the energy dissipative capability of hydrogen bonds (Figure 5c). With the addition of GO, the PUR/GO samples exhibited a slower stress relaxation rate in comparison with the virgin PUR sample. It was due to rigid GO hindering the conformational adjustment of the flexible rubber molecular chains. However, with the incorporation of MGO, the PUR/MGO samples presented a much faster stress relaxation rate compared to the virgin PUR sample. The relaxation rate gradually rose with the increase of molecular weight of PEG grafted on the surface of GO. The superior capability of the PUR/MGO system to dissipate stress energy also provided convincing evidence for the dissociation of hydrogen bonds during the deformation process.

To possess robust mechanical properties was the foremost performance toward engineering applications for all elastomeric materials. The mainly mechanical properties of the virgin PUR, PUR/GO and PUR/MGO composite membrane were shown in Figure 5d. For the virgin PUR vulcanizate, the tensile strength and elongation at break were 5.2 MPa and 420%. The tensile strength was improved over 2-fold increases after the incorporation of GO, but the elongation at break was decreased to 350% in the meantime. It indicated that the addition of GO brought a reinforcement of the mechanical strength but impaired the extensibility because the strong filler-rubber chain interaction inevitably hindered the mobility of elastomer network and then rigidified the flexible elastomer [7,36]. With the bionic design of multiple sacrificial hydrogen bonds into a DCP-cross-linked network, the PUR/MGO composite membranes exhibited a perfect improvement in strength and toughness after the incorporation of MGO. For example, the tensile strength was improved around 3-fold increases after the incorporation of MGO, and noteworthy was that the elongation at break was still larger than that of the virgin PUR vulcanizate. The present strategy was a practicable countermove to integrate strength and toughness of elastomer simultaneously.

What’s more, by regulating the structure parameters of multiple sacrificial hydrogen bonds (different molecular weight of PEG), the physical and mechanical properties of PUR/MGO varied. With the increase of molecular weight of PEG grafted on the surface of GO, the strength and toughness showed rose first and then fell. When the molecular weight of PEG was 600, the comprehensive performance was optimal. It should be attributed to the fact that the appropriate molecular weight of PEG selected for modifying GO would build an appropriate number of hydrogen bonds in the rubber matrix. The dissociation of these sacrificial hydrogen bonds would dissipate additional strain energy upon deformation. The hidden length hid in the filler-rubber interface and bound by the hydrogen bonds would be released, achieving the improvement of strength and toughness stimulatingly. Too small a molecular weight of PEG could not build enough hydrogen bonds, and too large was not enough to optimally increase the strength and toughness of the material. Because too long a molecular chain would curl into agglomerates, resulting in a reduced number of bonds that could be used to generate hydrogen bonds, and would result in a reduction in effective GO content at the same filler addition. In light of the above experiments, the incorporation of MGO-600 into PUR had been displayed to be a facile yet efficient strategy to implement energy dissipation mechanism toward robust elastomeric materials integrating strength and toughness.

### 3.6. Dispersion of MGO in PUR Matrix and Interaction with PUR Matrix

**Figure 6:**
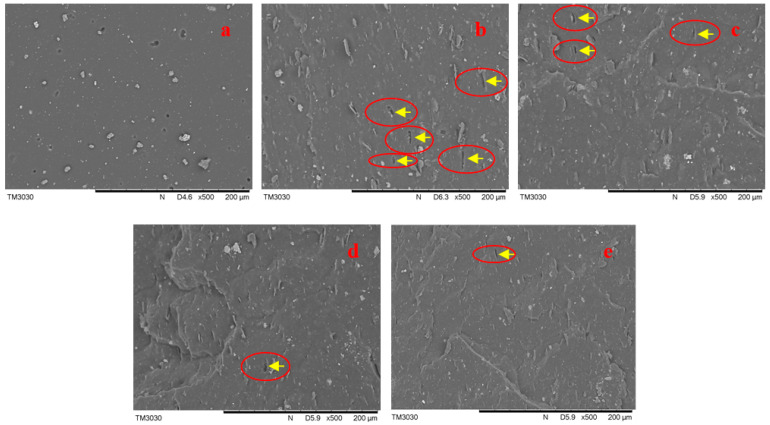
SEM analyses of PUR, PUR/GO and PUR/MGO. (**a**) Virgin PUR; (**b**) PUR/GO; (**c**) PUR/MGO-200; (**d**) PUR/MGO-600; (**e**) PUR/MGO-1000.

The dispersion of filler in PUR matrix and interaction with PUR matrix were characterized by SEM. The surface of virgin PUR sample (Figure 6a) was relatively smooth because on filler was added. The surface of PUR/GO sample displayed a slight folded and wrinkled texture (Figure 6b), and some rectangular holes could be seen on the surface (as indicated by the yellow arrows and red circle in Figure 6b). The reason for the appearing of these holes was that the agglomerated graphene was pulled out during the stretching process. The unmodified graphene had poor compatibility with the rubber matrix, it would agglomerate when it was added into the rubber matrix [33]. Compared with PUR/GO sample, the surfaces of PUR/MGO samples were rougher, and more wrinkle could be seen (Figure 6c–e). Furthermore, the interface between MGO and PUR was ambiguous, it indicated the interaction between MGO and PUR was better. The holes (as indicated by the yellow arrows and red circle in Figure 6c–e) were reduced because the dispersion of the filler in the rubber matrix was improved, and the agglomeration of the filler was significantly reduced when the filler was modified. In addition, Fewer GO could be found in the surface of PUR/MGO-1000 than that of PUR/MGO-600. It was due to the effective GO content of MGO-1000 was less than that of MGO-600 when the amount of added filler was the same mass, because more PEG was grafted on the GO surface. This was one of the main reasons why the mechanical properties of PUR/MGO-1000 were worse than that of PUR/MGO-600.

## 4. Conclusions

Imitating the robust structural strategy of natural biomaterials, different sacrificial hydrogen bond and hidden length were constructed in PUR/MGO composite membrane by regulating the different molecular weight of PEG used to modify GO. Appropriate number of hydrogen bonds constructed in the matrix is important for material properties. When the molecular weight of PEG was 600, the comprehensive performance of PUR/MGO composite membrane was optimal. A 223.1% increased in the tensile strength and an 11.9% increased in the elongation at break compared with the neat PUR. It was due to the energy dissipation and the release of hidden length because of the dissociation of the hydrogen bonds. Prolonging the storage time of PUR/MGO composites was beneficial for the recovery of the hydrogen bonds. Besides the hydrogen bonds, the great dispersion of GO in PUR matrix and compatibility with PUR matrix were also an important reason for the performance improvement of PUR/MGO composites. In addition, compared with PUR/GO composite membrane, T_max_s of PUR/MGO-200, PUR/MGO-600, PUR/MGO-1000 composite membranes increased by 4.0 °C, 5.9 °C, 5.4 °C and T_t_s of PUR/MGO-200, PUR/MGO-600, PUR/MGO-1000 composite membranes increased by 6.2 °C, 13.2 °C, 12.7 °C, respectively. T_g_s of PUR/MGO-600 and PUR/MGO-1000 composite membrane decreased by 4.5 °C and 7.2 °C compared with PUR/GO composite membrane.

## Figures and Tables

**Table 1:** The C and O element contents of GO and MGO.

Sample	GO	MGO-200	MGO-600	MGO-1000
C/%	92.56	83.86	70.09	68.86
O/%	7.44	16.14	29.91	31.14
O/C	0.08	0.19	0.43	0.45

**Table 2:** Vulcanization properties of PUR, PUR/GO and PUR/MGO.

Sample	Scorch Time (t_10_)/s	Curing Time (t_90_)/s	Minimum Torque M_L_/(dN·m)	Maximum Torque M_H_/(dN·m)	M_H_-M_L_/(dN·m)
Virgin PUR	40	657	0.37	17.81	17.44
PUR/GO	45	682	0.87	22.52	21.65
PUR/MGO-200	40	658	0.78	23.87	23.09
PUR/MGO-600	39	660	0.79	25.98	25.19
PUR/MGO-1000	41	661	0.76	27.09	26.33

**Table 3:** Key data of thermogravimetric analysis of PUR, PUR/GO and PUR/MGO.

Sample	T_i_ (°C)		T_max_ (°C)	T_t_ (°C)
	T_i_ of Platform A	T_i_ of Platform B		
Virgin PUR	458.6		462.1	472.5
PUR/GO	465.2		468.7	475.0
PUR/MGO-200	350.6	456.6	472.7	481.2
PUR/MGO-600	347.3	456.5	474.6	488.2
PUR/MGO-1000	344.7	456.0	474.1	487.7

## Data Availability

Not applicable.

## References

[1] Hagita K. (2018). Effect of diameter distribution on two-dimensional scattering patterns of a rubber model filled with carbon black and silica NPs. Polymer.

[2] Itskov M., Darabi E. (2016). Constitutive modeling of carbon nanotube rubber composites on the basis of chain length statistics. Compos. Part B Eng..

[3] Liu B., Wang S., Liu J., Tang Z., Guo B. (2019). Promoted strain-induced crystallization of cis-1,4-polyisoprene with functional carbon nanodots. Adv. Ind. Eng. Polym. Res..

[4] Suominen M., Damlin P., Granroth S., Kvarnström C. (2018). Improved long term cycling of polyazulene/reduced graphene oxide composites fabricated in a choline based ionic liquid. Carbon.

[5] Law Y.N., Hoey S.C., Ching Y.N. (2021). Incorporation of graphene oxide-based nanocomposite in the polymeric membrane for water and wastewater treatment: A review on recent development. J. Environ. Chem. Eng..

[6] Wu C., Huang X.Y., Wang G.L., Wu X., Yang K., Li S., Jiang P. (2012). Hyperbranched-polymer functionalization of graphene sheets for enhanced mechanical and dielectric properties of polyurethane composites. J. Mater. Chem..

[7] She X.D., He C.Z., Peng Z., Kong L. (2014). Molecular-level dispersion of graphene into epoxidized natural rubber, Morphology, interfacial interaction and mechanical reinforcement. Polymer.

[8] Keten S., Xu Z., Ihle B., Buehler M.J. (2010). Nanoconfinement controls stiffness, strength and mechanical toughness of β-sheet crystals in silk. Nat. Mater..

[9] Launey M.E., Buehler M.J., Ritchie R.O. (2010). On the mechanistic origins of toughness in bone. Annu. Rev. Mater. Sci..

[10] Gautieri A., Buehler M.J., Redaelli A. (2009). Deformation rate controls elasticity and unfolding pathway of single tropocollagen molecules. J. Mech. Behav. Biomed. Mater..

[11] Mao Y.Y., Wen S.P., Chen Y.L., Zhang F., Panine P., Chan T.W., Zhang L., Liang Y., Liu L. (2013). High performance graphene oxide based rubber composites. Sci. Rep..

[12] Tang Z.H., Huang J., Guo B.C., Zhang L., Liu F. (2016). Bioinspired engineering of sacrificial metal-ligand bonds into elastomers with supramechanical performance and adaptive recovery. Macromolecules.

[13] Zhao Z., Li L., Shao X.M., Liu X., Zhao S., Xie S., Xin Z. (2018). Tannic acid-assisted green fabrication of functionalized graphene towards its enhanced compatibility in NR nanocomposite. Polym. Test..

[14] Berki P., Hai L.N., Tung N.T., Karger-Kocsis J. (2018). Interphase tailoring via π-cation interaction in graphene and graphene oxide containing NR nocomposites prepared by latex compounding. Polym. Test..

[15] Yin B., Wang J.Y., Jia H.B., He J., Zhang X., Xu Z. (2016). Enhanced mechanical properties and thermal conductivity of styrene-butadiene rubber reinforced with polyvinylpyrrolidone-modified graphene oxide. J. Mater. Sci..

[16] Fantner G.E., Oroudjev E., Schitter L.S., Golde L.S., Thurner P., Finch M.M., Turner P., Gutsmann T., Morse D.E., Hansma H. (2006). Sacrificial bonds and hidden length: Unraveling molecular mesostructures in tough materials. Biophys. J..

[17] Lieou C.K.C., Elbanna A.E., Carlson J.M. (2013). Sacrificial bonds and hidden length in biomaterials- a kinetic constitutive description of strength and toughness in bone. Phys. Rev. E.

[18] Fantner G.E., Hassenkam T., Kindt J.H., Weaver J.C., Birkedal H., Pechenik L., Cutroni J.A., Cidade G.A.G., Stucky G.D., Morse D.E. (2005). Sacrificial bonds and hidden length dissipate energy as mineralized fibrils separate during bone fracture. Nat. Mater..

[19] Adams J., Fantner G.E., Fisher L.W., Hansma P.K. (2008). Molecular energy dissipation in nanoscale networks of Dentin Matrix Protein 1 is strongly dependent on ion valence. Nanotechnology.

[20] Rief M., Fernandez J.M., Gaub H.E. (1998). Elastically coupled two-level systems as a model for biopolymer extensibility. Phys. Rev. Lett..

[21] Huang J., Zhang L.J., Tang Z.H., Wu S., Ning N., Sun H., Guo B. (2017). Bioinspired design of a robust elastomer with adaptive recovery via triazolinedione click chemistry. Macromol. Rapid Commun..

[22] Song P.A., Xu Z.G., Lu Y., Guo Q. (2015). Bio-inspired hydrogen-bond cross-link strategy toward strong and tough polymeric materials. Macromolecules.

[23] Song P.A., Xu Z.G., Dargusch M.S., Chen Z.-G., Wang H., Guo Q. (2017). Granular nanostructure, a facile biomimetic strategy for the design of supertough polymeric materials with high ductility and strength. Adv. Mater..

[24] Zhang X., Liu W.F., Yang D.J., Qiu X. (2019). Biomimetic supertough and strong biodegradable polymeric materials with improved thermal properties and excellent UV-blocking performance. Adv. Funct. Mater..

[25] Luo D., Wang F., Vu B.V., Chen J., Bao J., Cai D., Willson R.C., Ren Z. (2018). Synthesis of graphene-based amphiphilic Janus nanosheets via manipulation of hydrogen bonding. Carbon.

[26] Kandhol G., Wadhwa H., Chand S., Mahendia S., Kumar S. (2019). Study of dielectric relaxation behavior of composites of Poly(vinyl alchohol)(PVA) and reduced graphene oxide(PGO). Vacuum.

[27] Liu X., Kuang W.Y., Guo B.C. (2015). Preparation of rubber/graphene oxide composites with in-situ interfacial design. Polymer.

[28] Sun H., Xu G.L., Xu Y.F., Sun S.-G., Zhang X., Qiu Y., Yang S. (2012). A composite material of uniformly dispersed sulfur on reduced graphene oxide, Aqueous one-pot synthesis, characterization and excellent performance as the cathode in rechargeable lithium-sulfur batteries. Nano Res..

[29] Bokobza L., Bruneel J.L., Couzi M. (2014). Raman spectroscopy as a tool for the analysis of carbon-based materials (highly oriented pyrolitic graphite, multilayer graphene and multiwall carbon nanotubes) and of some of their elastomeric vulcanizates. Vib. Spectrosc..

[30] Zirnstein B., Tabaka W., Frasca D., Schulze D., Schartel B. (2018). Graphene/hydrogenated acrylonitrile-butadiene rubber nanocomposites, Dispersion, curing, mechanical reinforcement, multifunctional filler. Polym. Test..

[31] Zhong X.O., Hanafi I., Azhar A.B. (2013). A comparative study of aging characteristics and thermal stability of oil palm ash, silica, and carbon black filled natural rubber vulcanizates. J. Appl. Polym. Sci..

[32] Potts J.R., Dreyer D.R., Bielawski C.W., Ruoff R.S. (2011). Graphene-based polymer nanocomposites. Polymer.

[33] Mao N.D., Jeong H., Nguyen T.K.N., Do T.V.V., Thuc C.N.H., Perré P., Ko S.C., Kim H.G., Tran D.T. (2019). Polyethylene glycol functionalized graphene oxide and its influences on properties of poly(lactic acid) biohybrid materials. Compos. Part B Eng..

[34] Lian H., Li S., Liu K., Xu L., Wang K., Guo W. (2011). Study on modified graphene/butyl rubber nanocomposites I Preparation and characterization. Polym. Eng. Sci..

[35] Roozbeh D., Mikhail I. (2009). A network evolution model for the anisotropic Mullins effect in carbon black filled rubbers. Int. J. Solids Struct..

[36] Bhattacharyya S., Sinturel C., Bahloul O., Saboungi M.-L., Thomas S., Salvetat J.-P. (2008). Improving reinforcement of natural rubber by networking of activated carbon nanotubes. Carbon.

